# Treatment and control of *Haemaphysalis longicornis* infestations on dogs using a formulation of sarolaner, moxidectin and pyrantel (Simparica Trio®)

**DOI:** 10.1186/s13071-025-06747-6

**Published:** 2025-03-26

**Authors:** Kristina Kryda, Masaya Naito, Takeshi Fujii, Andrew Hodge, Steven Maeder

**Affiliations:** 1 Zoetis, Veterinary Medicine Research and Development, 333 Portage St, Kalamazoo, MI 49007 USA; 2 Shokukanken Inc, 561-21 Arakuchi-Machi, Maebashi, Gunma 379-2107 Japan; 3 Zoetis Japan Inc, 3-22-7, Yoyogi, Shibuya-Ku, Tokyo, 151-0053 Japan; 4 Zoetis Australia Research and Manufacturing, Melbourne, Australia

**Keywords:** Asian longhorned tick, Canine, Efficacy, Isoxazoline, Oral combination, Prevention, Simparica Trio^®^

## Abstract

**Background:**

Simparica Trio^**®**^ (Zoetis), an orally administered combination product for dogs containing sarolaner, moxidectin and pyrantel pamoate, was evaluated against *Haemaphysalis longicornis*, a tick species originally native to Asia but now found on multiple continents, including North America.

**Methods:**

Two groups of eight dogs each were ranked based on pretreatment tick counts and then allocated through randomization to treatment on Day 0 with a single dose of either placebo or Simparica Trio at the minimum label dose of 1.2 mg/kg sarolaner, 24 µg/kg moxidectin and 5 mg/kg pyrantel (as pamoate salt). Dogs were infested with 50 viable adult *H. longicornis* on Days −2, 5, 12, 19, 26 and 33. Tick counts were conducted for all dogs 48 h after treatment and subsequent re-infestations.

**Results:**

Simparica Trio was 98.9% effective in treating existing *H. longicornis* infestation when considering live attached (feeding) ticks. Efficacy remained > 98% in preventing re-infestation for at least 35 days, and the geometric mean live attached tick counts for Simparica Trio-treated dogs were significantly lower than for placebo-treated dogs (*P* < 0.0001) at all timepoints assessed in the study. Dogs treated with Simparica Trio also had significantly lower (*P* < 0.0001) geometric mean total live (attached and free) tick counts than placebo-treated dogs at all times. No adverse events were reported for any dogs throughout the duration of the study.

**Conclusions:**

A single administration of Simparica Trio at minimum label dose was efficacious in treating and controlling adult *H. longicornis* in dogs for more than one month.

**Graphical Abstract:**

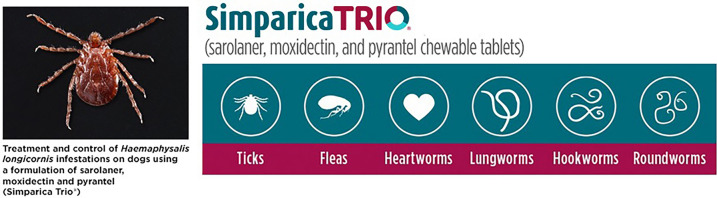

**Supplementary Information:**

The online version contains supplementary material available at 10.1186/s13071-025-06747-6.

## Background

The three-host tick *Haemaphysalis longicornis* (Acari: Ixodidae; Asian longhorned tick; cattle tick; bush tick) is prevalent throughout the Asia Pacific region, being native to Japan, Korea, eastern China and southeast Russia and an established introduced species in Australia, New Zealand and several islands in the Pacific [1–5]. In 2017, *H. longicornis* infestation was also reported from a farm in the USA, with many ticks recovered from a sheep in New Jersey [6]. Within 12 months, new populations were identified within the state of New Jersey as well as in New York, North Carolina, West Virginia, Virginia and Arkansas [7], and retrospective analyses of collected samples revealed *H. longicornis* in West Virginia as early as 2010 [7]. The highly invasive tick species continues to spread across the USA and has now been confirmed in 19 states [8]. The proficiency with which *H. longicornis* is able to rapidly infiltrate and establish in new areas is due largely to its non-specific host preferences, its parthenogenetic reproduction and its ability to survive and reproduce across a breadth of environmental temperatures [6, 9, 10]. Modeling suggests large areas of habitat around the world are suitable for the establishment of *H. longicornis*, including countries in which the tick has never been recorded. Suitable habitats in North America extend along the East Coast from Arkansas-South Carolina to Southern Quebec-Nova Scotia and along the West Coast from California to British Columbia [11, 12], and additional potential exists for establishment in Mexico and Central America [13], Africa and most European countries [4].

The global expansion of tick species over the years has paralleled an increase in tick-borne diseases [14]. Multiple pathogen species of concern to both animal and human health have been isolated from *H. longicornis,* including species of *Anaplasma*, *Borrelia*, *Babesia*, *Ehrlichia*, *Rickettsia* and *Theileria* as well as the causative agent of Q fever (*Coxiella burnetii*) [4, 15–18]. In some areas of Asia, *H. longicornis* is thought to act as vector for the virus that causes severe fever with thrombocytopenia syndrome in humans [19–21], an emerging disease of considerable concern in China because of its increasing incidence and high mortality rate [20]. The invasive expansion of *H. longicornis* into new geographic locales brings with it the potential introduction of novel pathogens to local hosts. One apparent example of this is the situation facing some cattle producers in the USA, who previously dealt only with nonpathogenic native *Theileria orientalis* genotypes but now must manage disease and resulting economic loss caused by the *T. orientalis* Ikeda genotype transmitted by recently invasive *H. longicornis* populations [22, 23].

While cattle are generally recognized as the dominant primary host for *H. longicornis*, additional hosts include other livestock (sheep, goats), companion animals (dogs, cats), wild mammals (deer, foxes, rabbits), and birds [2, 6, 10, 24–27]. One review of recorded *H. longicornis* infestations reports 77 different host species across eight countries, including many animals native to each country affected [4]. Dogs positive for *H. longicornis* have been identified in China, Japan, South Korea, Australia and the USA [4], with *H. longicornis* being the most frequently identified canine tick species in Japan [27] and increasingly found on dogs in Australia [28]. In the USA, where *H. longicornis* is a relatively new invasive species, dogs comprise the majority (85%) of reported companion animal infestations [7]. In parts of Asia, *H. longicornis* has long been considered an important vector of *Babesia gibsoni*, a protozoan parasite that can induce a spectrum of clinical symptoms in dogs [29–32]. In Australia, invasive *H. longicornis* has also been implicated in the spread of *B. gibsoni*, which was not identified in Australian dogs until 2002 [28, 33]. Similarly, as the incidence of canine *B. gibsoni* infections in the USA continues to rise [34, 35], it is not unreasonable to conjecture that invasive *H. longicornis* populations may become integrated in the life cycle of this pathogen.

Protection of dogs from *H. longicornis* infestation and the subsequent transmission of disease agents requires a parasiticide that will act rapidly to interrupt infestation and subsequent feeding. Additionally, to promote the greatest possible owner compliance, a product should be easy to administer and possess sustained efficacy, preferably against a broad spectrum of target parasites [36]. The past 10 years has seen the emergence of a series of novel parasiticides from the isoxazoline drug class highly effective in protecting dogs against multiple tick and flea species [37–40]. Sarolaner (Simparica^®^, Zoetis, NJ, USA), an isoxazoline approved for oral administration to dogs, has demonstrated efficacy against many tick species worldwide [41–45], including *H. longicornis* [46]. Simparica Trio^®^ is a monthly chewable preventative that combines sarolaner with moxidectin and pyrantel pamoate, allowing the convenient oral delivery of these three molecules from distinct drug classes as a single-dose means of treating and controlling important internal and external parasites in dogs [47]. This study investigated the efficacy of Simparica Trio administered once at minimum label dose [1.2 mg/kg sarolaner, 24 µg/kg moxidectin and 5 mg/kg pyrantel (as pamoate salt)] to treat an existing adult *H. longicornis* infestation and prevent weekly re-infestations for 5 weeks.

## Methods

The placebo-controlled, randomized, laboratory comparative efficacy study was conducted by Shokukanken Inc. (Gunma, Japan). All study procedures were in accordance with the World Association for the Advancement of Veterinary Parasitology guidelines for evaluating the efficacy of parasiticides for the treatment, prevention and control of flea and tick infestation on dogs and cats [48].

### Animals

Uniquely identified purpose-bred Beagle dogs (8 males, 8 females) aged 21 to 25 weeks and weighing 5.6 to 8.0 kg were included in the study. Detailed demographic data of dogs according to treatment allocation are shown in Supplementary Table 1. At enrollment, a veterinarian ensured all dogs were sufficiently healthy for study inclusion. Dogs had no history of previous ectoparasitic treatment or other therapies, except for a rabies vaccination administered 21 days before their enrollment on the study. Animals were housed in individual indoor enclosures such that physical contact was not possible with other dogs and were maintained at 18 to 29 ºC, in alignment with accepted animal welfare guidelines. Acclimatization to these conditions occurred for at least 10 days prior to the study starting on Day -7. Throughout the study, dogs were fed an appropriate ration of a commercial dry laboratory canine feed and were given water ad libitum. General health observations were performed daily for each dog starting on Day -17 through the duration of the study.

### Design

A randomized complete block design was employed. Prior to inclusion in the study, all 16 dogs were infested on Day -7 with 50 viable, unfed adult *H. longicornis* ticks to determine their host suitability. After 48 ± 2 h, live attached ticks were counted and removed. All 16 dogs were determined to be suitable for the study and were ranked into blocks of two based on pre-treatment tick counts. Two dogs within each block were then randomly allocated to treatment with either placebo or Simparica Trio with each treatment group containing eight dogs. Dogs were weighed and infested with 50 viable adult ticks on Day -2. On Day 0, all dogs underwent health assessments and were then given either the placebo or Simparica Trio. Tick infestations of 50 viable *H. longicornis* adults were repeated on Days 5, 12, 19, 26 and 33, and live tick counts were performed 48 ± 2 h after treatment on Day 0 and again after each infestation (on Days 7, 14, 21, 28 and 35).

### Treatment

All dogs were treated on Day 0 with either placebo tablets (inert formulation ingredients) or Simparica Trio chewable tablets, with doses calculated using body weights recorded on Day -2. Each dog in the Simparica Trio group received either one or two tablets of the combination product to be as close as possible to the minimum label dosage of 1.2 mg/kg sarolaner, 24 µg/kg moxidectin and 5 mg/kg pyrantel (as pamoate salt). Dogs in the placebo group received the equivalent number of placebo tablets. Both the placebo and Simparica Trio tablets were similar in appearance, and dogs were dosed by hand to ensure accurate pilling. Observation of each dog for several minutes after dosing confirmed all doses were swallowed. The prandial state of least absorption was utilized, with dogs not being fed within 12 h prior to dosing and for at least 4 h after treatment.

### Tick infestation and assessment

The study used viable, unfed adult *H. longicornis* ticks sourced from a laboratory colony first established at the laboratories of Shokukanken within 1 year of the study being conducted using wild engorged ticks collected in the Gunma Prefecture, Japan. At each infestation, *H. longicornis* adult ticks were placed only on the auricle to mimic the natural preferred attachment sites and were then allowed to freely move across the host’s body. Infestation chambers were not utilized. To facilitate infestation, dogs were sedated with xylazine hydrochloride (2% injectable formulation) administered subcutaneously at 2 mg/kg body weight. Additionally, grooming activities of each dog were inhibited through the fitting of Elizabethan collars. To ensure their host suitability and for allocation purposes, all dogs were first infested on Day -7 and live attached ticks were counted on Day -5. To determine efficacy against an existing infestation, dogs were then infested on Day -2 (2 days before treatment on Day 0) and tick counts were conducted on Day 2. To determine preventive efficacy duration, subsequent infestations were conducted weekly, on Days 5, 12, 19, 26 and 33, and counts were performed 48 ± 2 h after each infestation (on Days 7, 14, 21, 28 and 35).

### Tick counts

Dogs were sedated with xylazine hydrochloride (2% injectable formulation) administered subcutaneously at 2 mg/kg body weight at each tick count. Counts were conducted by trained personnel, and observed ticks were counted and removed. Dogs were systematically inspected from head to tail while parting the hair by hand and then were combed thoroughly with a fine-toothed comb. Each dog was examined for a minimum of 10 min, and if ticks were recovered during the last minute of counting, the examination was continued in 1-min increments until no ticks were recovered. All live and dead ticks were classified as either unattached or free. Ticks were removed from the dogs during each tick counting. Personnel conducting counts were masked to treatment assignments and changed gloves between dogs.

### Statistical analysis

The experimental unit was the individual dog. Tick counts were transformed using the log_e_(count + 1) transformation prior to analysis to decrease variance and normalize the data. Transformed counts were analyzed according to a mixed linear model for repeated measures using the PROC MIXED procedure (SAS 9.4, Cary NC). The fixed effects of the model were treatment, timepoint and the interaction between treatment and timepoint. The random effects included block, animal and error. Two-sided testing was performed at the significance level of α = 0.05.

Efficacy was evaluated separately for (i) total live tick counts (free and attached ticks) and (ii) live attached tick counts. Efficacy assessment was based on the percent reduction in the arithmetic and geometric mean live tick counts for Simparica Trio-treated dogs relative to those for placebo-treated dogs using Abbott’s formula:

% reduction = 100 × [mean count (placebo) – mean count (treated)]/mean count (placebo).

## Results and discussion

The 16 dogs enrolled in the study proved to be suitable hosts for *H. longicornis*, with Day -5 tick counts showing dogs retained between 26 and 46% of the 50 ticks administered on Day -7 [48]. Geometric mean (arithmetic mean; range) adult live tick counts were 17.1 (17.4; range 13 to 23). All eight placebo-treated dogs maintained robust adult tick infestations for the duration of the study. On Days 2, 7, 14, 21, 28 and 35, geometric mean live (free and attached) adult tick counts were 29.6, 26.8, 31.3, 27.9, 29.0 and 27.5, respectively, and arithmetic mean live (free and attached) tick counts were 29.8, 27.0, 31.5, 28.1, 29.0 and 27.5, respectively, for the placebo-treated group. More than 50% (range 51.1 to 63.3%) of the live adult ticks observed on placebo-treated dogs at each timepoint were attached. On Days 2, 7, 14, 21, 28 and 35, geometric mean live, attached adult tick counts were 16.6, 16.8, 15.7, 15.8, 17.4 and 16.6, respectively, and arithmetic mean live, attached tick counts were 16.9, 17.1, 16.1, 16.0, 17.6 and 16.8, respectively. A few dead ticks (0 to 2) were found on placebo-treated dogs at each timepoint.

Actual administered doses of Simparica Trio ranged from 1.23 to 1.58 mg/kg sarolaner, 25 to 32 µg/kg moxidectin and 5.14 to 6.58 mg/kg pyrantel (as pamoate salt). Dogs administered Simparica Trio displayed no adverse reactions to treatment during daily general health observations. On Days 2, 7, 14, 21, 28 and 35 post-treatment, arithmetic mean live (free and attached) tick counts in Simparica Trio-treated dogs were 8.8, 11.9, 9.1, 7.9, 10.9 and 7.1, respectively, and geometric mean live (free and attached) tick counts were 8.0, 11.6, 8.8, 7.3, 10.5 and 6.3, respectively. The eight dogs treated with Simparica Trio showed significantly reduced (*P* < 0.0001) geometric mean live (free and attached) adult tick counts compared to placebo-treated dogs at each timepoint examined (5.64 ≤ t(50.8) ≤ 9.62, *P* < 0.0001). In these same Simparica Trio-treated dogs, the number of killed adult ticks collected from each dog at each timepoint ranged from 14 to 27 (28 to 54% of the infestation). Almost all (98%) live adult ticks recovered from Simparica Trio-treated dogs were unattached, with geometric (arithmetic) mean live attached tick counts for Simparica Trio-treated dogs being ≤ 0.3 (≤ 0.4) at each timepoint (Table 1). A total of only nine live, attached adult ticks were recovered from the Simparica Trio-treated dogs over the 35 days: two dogs never had live attached ticks; three dogs were observed to have a single live, attached tick at one timepoint; three dogs were observed to have a single live, attached tick at two different timepoints.

**Table 1:** Efficacy of Simparica Trio in treating *Haemaphysalis longicornis* infestations and preventing subsequent weekly infestations compared to placebo at 48 h after treatment

Placebo				Simparica Trio^1^			Percent efficacy^2^
Day	Geometric mean (arithmetic mean)	Range	No. of dogs with ticks	Geometric mean (arithmetic mean)	Range	No. of dogs with ticks	
2	16.6 (16.9)	12–22	8/8	0.2 (0.3)	0–1	2/8	98.9 (98.5)
7	16.8 (17.1)	12–22	8/8	0.1 (0.1)	0–1	1/8	99.5 (99.3)
14	15.7 (16.1)	11–21	8/8	0.2 (0.3)	0–1	2/8	98.8 (98.5)
21	15.8 (16.0)	13–19	8/8	0.0 (0.0)	0–0	0/8	100 (100)
28	17.4 (17.6)	13–21	8/8	0.3 (0.4)	0–1	3/8	98.3 (97.9)
35	16.6 (16.8)	13–20	8/8	0.1 (0.1)	0–1	1/8	99.5 (99.3)

^1^Treatment with placebo or Simparica Trio [minimum dose 1.2 mg/kg sarolaner, 24 µg/kg moxidectin, 5 mg/kg pyrantel (as pamoate salt)] occurred on Day 0. ^2^Geometric (arithmetic) efficacy of Simparica Trio versus placebo. Geometric mean live, attached tick counts were significantly lower compared to placebo at each timepoint (22.66 ≤ t(71.6) ≤ 24.18, *P* < 0.0001)

It is logical to assume that live, unattached adult ticks recovered from dogs during this study were not feeding and thus had not been exposed to Simparica Trio. Therefore, efficacy of Simparica Trio against *H. longicornis* was calculated using the live, attached adult tick counts recorded for dogs in each treatment group (Table 1). In this study, geometric mean counts of live, attached adult ticks for Simparica Trio-treated dogs were significantly lower at each timepoint compared with placebo-treated dogs (22.66 ≤ t(71.6) ≤ 24.18, *P* < 0.0001, Table 1). One treatment with Simparica Trio provided dogs with 98.9% efficacy against existing adult *H. longicornis* infestations and was ≥ 98.3% effective in protecting dogs against re-infestation with adult *H. longicornis* for at least 35 days after treatment.

The impacts of tick infestations on dogs are well known [49, 50], and the negative consequences of *H. longicornis* infestation have been documented across multiple host species, ranging from irritation and anemia to diarrhea, production losses and even death [1, 10]. Laboratory studies on *H. longicornis* have demonstrated that all life stages can feed on dogs, with mean larval, nymphal and adult feeding periods similar to those recorded when feeding on cattle [1, 51, 52]. Additionally, environmental sampling has recovered host-seeking engorged *H. longicornis*, providing evidence for repeated successful questing events and highlighting its possible function in the spread of vector-borne agents [53]. The role of *H. longicornis* in transmitting human and animal diseases, the negative impacts associated with its infestations, its wide host range and its invasive nature show the dangers posed by this tick species. Previous studies show that a single oral dose of sarolaner can rapidly and effectively control multiple canine tick species [41, 42, 44, 45, 54–56], including *H. longicornis* [46]. Here, we show sarolaner administered in a combination product, as Simparica Trio, remained highly effective in rapidly treating existing adult attached, live *H. longicornis* infestations in dogs and in preventing re-infestation for at least 35 days. This level of protection interrupts *H. longicornis* development and decreases the likelihood of pathogen transmission between hosts.

When administering traditional pill or tablet medications, many dog owners worry about inaccurate dosing and damaging the animal-human bond during unsuccessful or stressful pilling events [57]. In contrast, dog owners generally view chewable medications positively because of their increased ease of administration and formulation palatability. The Simparica Trio chewable tablet has been shown to be well accepted by dogs, with one study reporting the majority (91.9%) of 517 doses offered to dogs by owners in their own homes were accepted either without food or in food (and only 8.1% of doses required physical administration) [58]. Moreover, as a combination product containing both moxidectin and pyrantel in addition to sarolaner, Simparica Trio provides dog owners seeking month-long efficacy against ticks and fleas the additional benefits of effective treatment for roundworm and hookworm infections and protection against lungworm and heartworm disease [56, 58–61]. When one considers the important roles of palatability, ease of administration and broad-spectrum protection in owner compliance [36, 57, 62], it is clear that Simparica Trio makes an important contribution in the field of canine health by providing comprehensive and convenient protection.

## Conclusions

Existing canine *H. longicornis* infestations were effectively treated with one oral administration of Simparica Trio at the minimum label dose of 1.2 mg/kg sarolaner, 24 µg/kg moxidectin and 5 mg/kg pyrantel (as pamoate salt). This medication provided effective treatment of *H. longicornis* adult infestations and prevented re-infestation for up to 35 days. Simparica Trio is an easy and convenient means of providing dogs with rapid protection against many important tick species.

## Supplementary Information

Supplementary materials 1.

## Data Availability

The dataset supporting the conclusions of this article is included within the article.
