# Supplementary material for: Treatment and control of Haemaphysalis longicornis infestations on dogs using a formulation of sarolaner, moxidectin and pyrantel (Simparica Trio®)
Source: Parasit Vectors. 2025 Mar 26;18:117. doi: 10.1186/s13071-025-06747-6 (PMC11948799; doi:10.1186/s13071-025-06747-6)
Supplement: Supplementary file 1 — Supplementary materials 1. [file 13071_2025_6747_MOESM1_ESM.docx]

**Supplementary Table 1**

Demographic characteristics of enrolled dogs according to treatment allocation

|  |  |  | **Placebo**^2^ | **Simparica Trio**^2^ | **Total** |
| --- | --- | --- | --- | --- | --- |
| **Sex**^1^ | **Female** | n | 3 | 5 | 8 |
|  |  | % | 37.5 | 62.5 | 50.0 |
|  | **Male** | n | 6 | 3 | 8 |
|  |  | % | 62.5 | 37.5 | 50.0 |
| **Age on Day 0**  **(weeks)** | | n | 8 | 8 | 16 |
|  |  | Mean | 23.5 | 23.3 | 23.4 |
|  |  | SD | 1.7 | 1.4 | 1.5 |
|  |  | Min | 21 | 21 | 21 |
|  |  | Max | 25 | 25 | 25 |
| **Body Weight on Day -2 (kg)** | | n | 8 | 8 | 16 |
|  |  | Mean | 7.23 | 7.35 | 7.29 |
|  |  | SD | 0.76 | 0.53 | 0.64 |
|  |  | Min | 5.6 | 6.3 | 5.6 |
|  |  | Max | 8.0 | 8.0 | 8.0 |

^1^All dogs were purpose-bred purebred beagles. ^2^Treatment with placebo or Simparica Trio (minimum dose 1.2 mg/kg sarolaner, 24 µg/kg moxidectin, 5 mg/kg pyrantel (as pamoate salt)) occurred on Day 0.
